# Algae-Derived Peptides as Functional Food Ingredients: Bioactivities, Processing Challenges, and Computational Design Strategies

**DOI:** 10.3390/foods15050811

**Published:** 2026-02-26

**Authors:** Keying Su, Juanjuan Ma, Qian Li, Xuewu Zhang, Laihoong Cheng

**Affiliations:** 1 College of Engineering, Guangzhou College of Technology and Business, Guangzhou 510850, China; 2 Food Technology Division, School of Industrial Technology, Universiti Sains Malaysia, Pulau Pinang 11800, Malaysia; 3 College of Food Science and Engineering, South China University of Technology, Guangzhou 510642, China

**Keywords:** algae-derived peptides, sustainable resources, structure–activity relationships, bioactive functionalities, computational technology, applications

## Abstract

Algae-derived proteins and peptides have gained increasing interest as sustainable bioresources with valuable nutritional and functional properties. This review aims to synthesize current knowledge on their characteristics and applications while highlighting the emerging role of computational tools in peptide research. Key findings show that algae provide diverse proteins and bioactive peptides with advantageous amino acid profiles and notable antioxidant, antihypertensive, antidiabetic, anti-inflammatory, and skin-protective activities. Their applications span food formulation, pharmaceuticals, and cosmetics, although large-scale utilization remains constrained by production, stability, and bioavailability challenges. Computational strategies, including virtual enzymatic hydrolysis, machine-learning prediction, QSAR modeling, molecular docking, molecular dynamics, and toxicity/allergenicity assessment, offer promising avenues for efficient peptide discovery, though their use in algae is still limited. Overall, this review underscores the potential of algae-derived proteins and peptides as multifunctional ingredients and emphasizes the need to integrate in silico pipelines with improved processing and delivery systems to accelerate future translational applications.

## 1. Introduction

Algae, encompassing both macroalgae and microalgae, are photosynthetic organisms that have garnered significant attention for their potential as sustainable protein sources. Algae are particularly rich in proteins and amino acids, with a protein concentration that can range from 47% to 65% of dry weight, which is useful for both human consumption and animal feed [1]. Algal proteins have been reported to possess antioxidant, antimicrobial, anti-inflammatory, antihypertensive, and antitumor properties, making them valuable for the nutraceutical, cosmetic, and pharmaceutical industries. In the food sector, they are used to enhance the nutritional value of products and improve sensory qualities without altering cooking or textural characteristics [2]. Given the observed bioactivities of peptides in regulating the immune, cardiovascular, nervous, and gastrointestinal systems, pharmacological interest in peptides and proteins as therapeutic agents is growing. Their biocompatibility, potency, and selectivity offer alternatives for the effective treatment of non-communicable diseases with fewer side effects [3]. In the cosmetics industry, bioactive compounds from algae are utilized for their skin-nourishing and anti-aging effects. Despite their potential, challenges persist in the application of algal proteins. Despite these advances, several important gaps arose in the broader translation of algae-derived proteins and peptides into safe, efficacious, and regulatory-compliant products. On the one hand, mechanistic and target-level evidence is often sparse. Many reported bioactivities are shown in vitro on crude fractions due to cost and time constraints without a molecular-level understanding of targets or modes of action. On the other hand, safety, toxicity and allergenicity data for novel algal peptides are incomplete, which slows translational development for food and therapeutic uses.

Many of these gaps can be substantially mitigated by a systematic integration of in silico approaches. In silico tools enable rapid prioritization and mechanistic evaluation of peptide candidates by integrating machine-learning activity prediction, molecular docking/MD-based interaction analysis, and ADMET/toxicity screening. These approaches streamline peptide discovery, reduce experimental costs, and enhance safety and efficacy assessment before in vitro or in vivo validation. By utilizing in silico methods, specific bioactive peptides can be identified from algal hydrolysates, enhancing the understanding of their structure-function relationships [4]. Bioinformatics tools have been used to predict potential peptides with diverse biological activities (e.g., DPP-IV inhibitors, ACE inhibitors, antioxidants) from *Chlorella* and other species of algae [5]. These tools enable researchers to explore and exploit the bioactive potential of algae-derived peptides for pharmaceutical, nutraceutical, and even dermal applications. Currently, the application of in silico technologies in peptide research remains at an early stage. Recent reviews emphasized that integrating advanced machine learning, molecular dynamics, and large-scale peptide databases could substantially improve accuracy and predictive power, but these strategies have not yet been fully exploited in food and nutraceutical peptide research [5]. Therefore, the untapped potential of in silico approaches offers significant opportunities to accelerate bioactive peptide identification, enhance mechanistic understanding, and guide translational applications.

In recent years, several reviews have summarized algal proteins, bioactive peptides, and their functional food applications, often focusing on specific species, extraction technologies, or reported bioactivities. While these contributions have significantly advanced the field, most remain centered on cataloguing activities or discussing technological processes in isolation. Limited attention has been given to systematically integrating mechanistic pathways, methodological heterogeneity, production scalability, regulatory feasibility, and emerging computational strategies within a unified framework.

In contrast, this review adopts a systems-oriented and forward-looking perspective. Beyond summarizing antioxidant, antihypertensive, antimicrobial, and immunomodulatory activities, we critically evaluate methodological variability, highlight key limitations in current experimental models, and connect bioactivity mechanisms with upstream biomass variability and downstream regulatory considerations. Importantly, the emerging convergence of traditional experimental validation with computational tools was emphasized, including molecular docking, QSAR modeling, and machine learning-assisted peptide prediction, as a strategy to enhance discovery efficiency and reduce empirical redundancy. By integrating mechanistic insight, production scalability, regulatory context, and data-driven screening approaches, this review aims to provide a translational roadmap that moves the field from descriptive bioactivity reporting toward precision-guided development of functional algal ingredients.

## 2. Algae-Derived Proteins and Peptides

### 2.1. Overview of Protein and Peptide Content in Different Types of Algae

Algae proteins are nutritionally valuable and encompass several enzymes, phycobiliproteins, lectins, protein-derived hydrolysates and peptides [6]. Glycoproteins (GPs) are proteins covalently linked to two main types of oligosaccharide chains with covalent bonds, by N-glycosyl linkages or by O-glycosyl linkages. They usually include a hexose sugar (often galactose or mannose, and less frequently glucose). The oligosaccharide chains are usually linked to proteins through glycosylation during co-translational modification or post-translational modification. GPs are normally found on the cell surface or free following release, and their functions comprise intercellular communication and identification [7]

Lectins are GPs purified from various plants and animals. Algae are good sources of novel lectins of low molecular weight, especially red algae [8]. Lectin helps agglutinate cells and form monosaccharide or polysaccharide complexes. It is vital in such signaling pathways as signal transduction and immune response. Plant lectins, commonly found as phytoagglutinin (PHA), concanavalin (ConA), wheat germ agglutinin (WGA), peanut agglutinin (PNA) and soybean agglutinin (SBA), have a wide range of functions including cell agglutination, antiviral and antifungal activity, and activation of apoptosis or autophagy [9,10,11]. Consequently, they have high research value and promising applications in life sciences, medicine, and agriculture. Currently, lectins have been discovered in some algae, and they have certain application potential, such as red algae like *Griffithsia* sp.; green algae like *Boodlea coacta*, *Bryopsis plumosa*; and brown algae like *Agardhiella subulate*, *Eucheuma amakusaensis*, *Eucheuma cottonii*, *Eucheuma denticulatum*, *Kappaphycus striatum*, etc.

Phycobiliprotein is composed of apoprotein and phycobilin (PCB) covalently linked through one or two thioether bonds. Types of phycobiliproteins known phycobiliproteins can be divided into the following four categories: phycoerythrin (PE), phycocyanin (PC), phycoerythrocyanin (PEC) and allophycocyanin (APC). Phycobiliprotein is an important light-harvesting protein unique to some algae. As early as the beginning of the last century, the existence of fluorescent red, violet, and blue proteins in red algae and cyanobacteria was reported [12]. Recent studies have demonstrated that phycobiliprotein can be utilized as a natural pigment in sectors such as food, cosmetics, and dyes, as well as to make fluorescent reagents for application in clinical medical diagnostics, immunochemistry, and bioengineering. Furthermore, they can be developed into functional foods and pharmaceuticals.

Season, maturity, species, and environmental variables affect the amount of protein, peptides, and amino acids in algae [13,14]. In the whole, algae contain up to 60% protein by dry weight, which is comparable to typical protein sources such as meat, eggs, soy products, and milk [15]. Soybeans, one of the richest protein products, have a protein content of about 12.59% [16]. At present, the algae studied by scholars are roughly divided into four categories: cyanobacteria, red algae, green algae, and brown algae. Red algae protein accounts for 7–47% of dry weight, green algae accounts for 9–33% of dry weight, and brown algae protein accounts for 3–24% of dry weight [15]. Protein content in cyanobacteria varies widely (2.5–73.7%), with an average concentration of 36.9% [17]. Table 1 summarizes the protein content of some edible seaweeds in four phyla.

### 2.2. Characteristics of Algae-Derived Proteins and Peptides

The quality of a protein can be evaluated based on its amino acid (AA) composition, digestibility, and bioavailability, which makes algae-derived proteins a valuable alternative source. Among these factors, AA composition is a critical indicator of protein nutritional value, as a higher proportion of essential amino acids (EAAs) contributes significantly to meeting human daily dietary requirements. Algae-derived proteins often contain substantial amounts of EAAs, with EAA content accounting for more than 30% of total protein composition in many species. Some algal species even exhibit EAA ratios exceeding 60% (Table 1). As shown in Table 1, red algae and cyanobacteria with high protein contents typically present EAA proportions ranging from 40% to 50%, except for *Porphyra* sp., which contains a notably high EAA content of 56.73%. In contrast, several green algae showed relatively stable EAA proportions around 40%, while *Chlorella* exhibited a slightly lower value of 33.2%. Interestingly, brown algae—despite having lower overall protein contents—tend to have higher EAA proportions, often exceeding 50% (e.g., *Fucus spiralis*).

According to the study [16], the AA composition of five algal species was analyzed, revealing that *Chlorella vulgaris* and *Dunaliella salina* exhibited almost identical profiles. Among the EAAs, leucine, valine, and phenylalanine are particularly abundant across all examined species, with leucine typically being the most prevalent. The richness of EAAs in certain algal proteins is comparable to that of conventional high-quality protein sources, such as legumes (~45%), ovalbumin (52.4%), and casein (43.6%) [34]. Furthermore, algal proteins often exhibit superior quantitative and qualitative profiles of EAAs compared to many plant- and animal-derived proteins. Algal amino acids are generally considered healthier, more digestible, and less toxic than synthetic counterparts, offering nutritional benefits for both humans and animals. However, the amino acid composition of many algal species remains underexplored. This lack of detailed knowledge poses a significant barrier to their broader application in feed additives and nutritional supplements. To overcome this limitation, further research is needed to characterize the amino acid profiles of various algae species and to develop efficient extraction and purification techniques for their bioactive components.

Net protein utilization (NPU) is a measure of the degree to which proteins are utilized by the body. It includes both aspects of food protein digestion and utilization, so it is more comprehensive for protein quality assignment. The higher the net protein utilization value, the better the quality of the protein. Protein utilization includes digestibility and absorption mechanisms, and studying protein utilization requires a combination of experiments in vivo and in vitro. In animal experiments, it is expressed as the percentage of the amount of nitrogen utilized in the body after digestion and absorption of protein to the amount of nitrogen [35]. However, animal tests cost a significant amount of time and money, and currently, the in vitro digestibility of algae protein is more commonly used for protein quality evaluation. Pepsin, trypsin, chymotrypsin, or a mixture of these enzymes is commonly used for in vitro simulated digestion of algal proteins, and the degree of hydrolysis of algal proteins is determined [36,37].

As shown in Table 1, the protein digestibility of various algal species ranges from 40% to 90%. Among them, red algae such as *Sarcodiotheca gaudichaudii* (85.7%) and *Palmaria palmata* (87.3%) exhibit relatively high digestibility, whereas brown algae like *Undaria pinnatifida* show lower values, with digestibility as low as 48.1%.

The in vitro digestibility varies greatly because of the specific assay methods and enzymatic hydrolysis conditions used. In addition, the types of glycoproteins and antinutritional factors and seasonal changes affect the algae protein digestibility [37]. This lack of consistency makes the resulting data difficult to compare directly. Furthermore, cell wall structure, disruption methods, and amino acid profiles all have an effect on algal protein digestibility and bioavailability [38,39]. Some strains have robust cell walls that limit protein digestibility, necessitating efficient disruption techniques to enhance the accessibility of digestive enzymes. Studies have shown that combined disruption methods like enzymatic pretreatment can increase the bioavailability of proteins and other bioactive compounds in algae, improving their absorption in the body [40]. Some authors proposed that heating the algae sample before simulated human ingestion may boost the bioaccessibility of protein/amino acids [41].

To sum up, the quality of protein is influenced by its amino acid (AA) composition, digestibility, and bioavailability, with algae offering a promising alternative protein source due to their high levels of essential amino acids (EAA). Red algae and cyanobacteria typically have EAA ratios of 40–50%. Brown algae, though lower in protein, often have high EAA content. Algae also contain significant amounts of leucine, valine, and phenylalanine, comparable to high-quality protein sources like legumes and casein. In terms of digestibility, algae protein can range from 40% to 90%. However, the in vitro digestibility of algae proteins varies due to different experimental methods, the presence of glycoproteins, antinutritional factors, and seasonal changes, which affect direct comparison between studies.

### 2.3. Potential Advantages over Traditional Protein Sources

Consuming animal proteins increases the risk of developing noncommunicable diseases such as cancer [42], heart disease [43], non-alcoholic fatty liver disease (NAFLD) [44], and inflammatory bowel disease (IBD) [45]. Furthermore, the global population is rapidly increasing, with an expected 9.8 billion people by 2050 [46]. Dietary protein demand is rising due to population growth, creating a supply concern. According to the Food and Agriculture Organization, over 821 million people worldwide are undernourished due to an insufficient protein-rich diet [47]. As a result, there is an increasing need to investigate alternative protein sources, such as plant proteins, to address the issues associated with traditional animal protein sources. In this context, microalgae have been identified as promising resources capable of producing a sustainable source of protein

Algae-derived proteins offer several advantages over traditional protein sources. Firstly, microalgae and seaweeds are rich in protein; some microalgae contain protein levels twice as high as traditional sources. Secondly, microalgae proteins have a well-balanced amino acid profile meeting human nutrition requirements and displaying high-quality characteristics. Additionally, algae-based proteins have shown potential health benefits, such as anti-inflammatory, antioxidant, and anti-cancer properties, and liver health promotion [48], making them ideal for functional food development. Moreover, algae cultivation combines bioengineering and chemical engineering, which is entirely apart from agriculture [49]. Taking *Spirulina* cultivated in raceway ponds as an example, the annual yield of *Spirulina* powder per mu (calculated as 60% protein content) exceeds 900 kg [50]. Microalgal production is environmentally friendly, low-carbon, or even negative-carbon production, without involving chemical fertilizers or pesticides.

Although more than 12,000 algal species have been recorded worldwide, only a limited number possess characteristics that make them suitable for peptide research and functional food applications. The species highlighted in this review, such as *A. platensis*, *Chlorella vulgaris*, and *Nannochloropsis oculate*, are characterized by relatively high protein content, favorable amino acid profiles, and well-documented bioactive potential. In addition, these species are commercially cultivated at large scale, allowing a consistent biomass supply and reproducible processing conditions. Their protein composition and biochemical properties have been extensively characterized, facilitating enzymatic hydrolysis and peptide identification.

## 3. Applications of Algae-Derived Proteins and Peptides

### 3.1. Food and Beverage Industry

These proteins can be utilized in various food applications such as the bakery industry, dairy industry, beverage industry and snack foods to boost their protein content and nutritional value [47]. For example, *Spirulina* and *Chlorella* proteins are commonly used in protein-enriched beverages, such as smoothies and shakes, providing essential amino acids and enhancing protein intake for athletes and health-conscious consumers [51]. Additionally, microalgae have been integrated into baked goods, including bread and biscuits, where they not only improve protein content but also contribute to dough rheology, increasing water absorption and improving texture in the final production [52]. Algae-derived peptides possess antioxidant properties that can enhance the shelf life of baked products by delaying lipid oxidation, which is a key factor in the spoilage of fats in such foods. Algal proteins have also been explored as ingredients in plant-based meat alternatives due to their high protein content and functional properties, like emulsification and water retention [53,54]. For example, *Chlorella* and *Nannochloropsis* proteins have been used to improve the texture of plant-based burgers and sausages, helping to achieve the fibrous, chewy texture typical of meat [55]. Algae-derived peptides have also found applications in dairy products, such as yogurt and cheese, as bioactive compounds that enhance health benefits through antioxidant and antihypertensive properties [52]. In snacks, algae-derived components are incorporated into protein bars and crisps, adding both nutritional value and distinctive umami flavor [56]. Furthermore, algae proteins are increasingly used in pasta and noodles to improve their nutritional profile, providing plant-based protein options [57,58]. Algal proteins are also utilized in soups, sauces, and spreads for their rich protein content and functional attributes like thickening and stabilization [59].

While algae-derived proteins and peptides offer considerable benefits for enhancing the nutritional and functional properties of foods, there are challenges related to sensory attributes and processing that need to be solved. One of the primary challenges is their impact on taste, color, and aroma. Algal ingredients often impart a distinct green hue and a “marine” flavor, which may not be desirable in conventional foods. Moreover, the extraction and purification processes for algae proteins can be relatively expensive, which may increase the cost of food products compared to traditional technology processes [55,59].

Microalgae-derived pigments, such as chlorophylls, carotenoids, and phycobiliproteins, are gaining appeal in the food business due to their distinct hues, molecular structures, and physiological properties [60]. Phycobiliproteins (PBPs) found in algae are intense color pigment-proteins that can serve as natural food colorants [61]. These natural colorants derived from marine algae provide not only bright hues but also health advantages such as antioxidants and anti-diabetics [62]. For instance, the blue-green pigment phycocyanin from *Arthrospira platensis* is used in beverages [63], from *A. platensis* is used in soft candy [64], and from *Porphyridium cruentum* is used in milk-based products [65]. Similarly, the red pigments, phycoerythrin, from such algae as *Porphyridium* spp., *Gracilaria* spp., *Chondrus* spp., and *Hypnea* spp. have been explored as a natural red colorant in various food applications, including confectionery, dairy products, and sauces. A study by Hsieh et al. [66] shows that phycoerythrin can effectively replace synthetic red colorants in sweets and candies, offering both color and nutritional advantages. In yogurt and other dairy products, phycoerythrin has been utilized to enhance color while maintaining product stability. It was demonstrated that incorporating phycoerythrin into yogurt not only improved the visual appeal but also contributed antioxidative properties, making it a desirable ingredient for health-focused formulations [67]. Phycoerythrin’s stability in acidic conditions makes it suitable for use in sauces. Research found that phycoerythrin retains its color integrity and bioactivity even in the acidic environment of sauces, thus providing an attractive and functional colorant for this food category [68].

Although microalgae proteins exhibited good bioactivity, protein extraction cost a lot of time and a large amount of money, and biomass powder of algae seems to be a more economical choice. Algae biomass powder was applied as a functional food ingredient in many kinds of food, particularly concerning the formulation of fortified foods, nutritional shakes, bread, snacks, cookies, pasta, cereal bars, instant soup, pudding, cake powder mix, and isotonic beverages [69,70].

The most commonly used are *spirulina* and *chlorella*. *Spirulina* has been incorporated into a range of products, including biscuits, pasta, dairy products, and ice cream, to enhance their nutritional value. For instance, the addition of spirulina to biscuits has been shown to increase protein content and overall nutritional value [70]. Similarly, the incorporation of *spirulina* in pasta has led to an increase in protein and energy content, making it a more nutritious option [70]. The use of *spirulina* in dairy products, such as yogurt and ice cream, has also been explored, with positive results in terms of improving protein content and antioxidant activity [70]. According to research [71], integrating *Tetraselmis* spp., *Spirulina* spp., and *Chlorella* spp. into broccoli soup resulted in a significant increase in antioxidant activity. This increased antioxidant activity may be due to microalgae’s higher PC. During simulated gastrointestinal digestion, these proteins may have undergone hydrolysis, leading to the release of bioactive peptides with antioxidant capabilities. A study [72] found that the incorporation of microalgae in a vegetable cream resulted in a product with significantly higher protein content and antioxidant capacity, with no significant differences in sensory acceptability compared to the control. Some relative studies were listed in Table 2.

In conclusion, the integration of microalgae-derived protein and peptides into functional foods offers a nutritious and health-promoting alternative to traditional ingredients. The data from the studies reviewed here indicate that microalgae can significantly enhance the protein content, antioxidant properties, and health benefits of food products, making them a valuable component in the development of functional foods for human consumption.

### 3.2. Pharmaceutical Industry

Algae-derived proteins and peptides exhibit pharmacological potential for drug development [87,88]. These compounds possess anti-inflammatory, antioxidant, and neuroprotective properties, making them valuable in treating various diseases [89,90]. Studies have shown that peptides from microalgae, when stabilized using carriers like gold nanoparticles, can enhance their stability and efficacy in biological systems [91].

**Figure 1:**
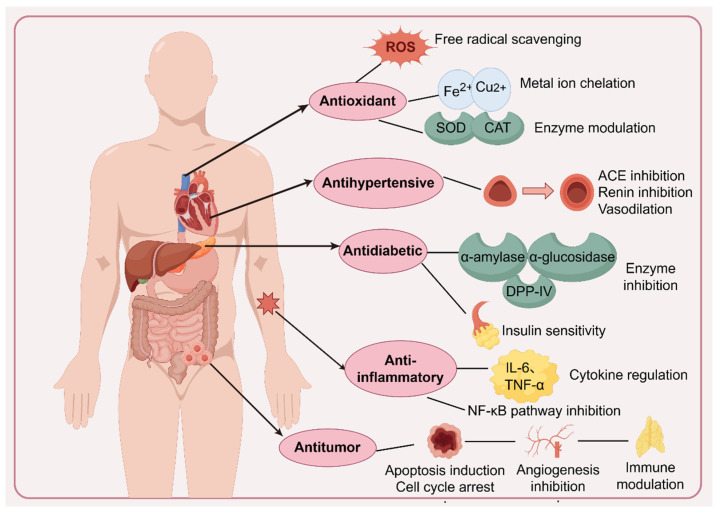
Common mechanisms of algae bioactive peptides.

Figure 1 illustrates the common mechanisms of algae-derived bioactive peptides and summarizes their multifaceted physiological effects at the molecular, cellular, and systemic levels. As shown in the figure, these peptides exert biological functions through several interconnected pathways, including antioxidant, antihypertensive, antidiabetic, anti-inflammatory, and antitumor activities. At the molecular level, antioxidant effects are achieved through direct scavenging of reactive oxygen species (ROS), chelation of pro-oxidant metal ions such as Fe^2+^ and Cu^2+^, and modulation of endogenous antioxidant enzymes, including superoxide dismutase (SOD) and catalase (CAT). These mechanisms contribute to the mitigation of oxidative stress, which underlies many chronic diseases. Many studies focus on the significant antioxidant properties of algal proteins and peptides, which are crucial in combating free radicals and reactive oxygen species (ROS) that can lead to various health issues, including cancer. Specifically, peptides containing hydrophobic amino acids like Phe, Trp, Tyr, Ala, Val, and Leu contribute to free radical scavenging by facilitating membrane lipid bilayer penetration [92,93]. Basic or acidic amino acids, including Lys, Asp, and Glu, can chelate metal ions, while aromatic amino acids quench free radicals through direct electron transfer [94].

In cardiovascular regulation, antihypertensive activity is primarily associated with inhibition of ACE and renin, as well as the promotion of vasodilation, thereby contributing to blood pressure control. For instance, proteins from *Arthrospira platensis* have shown potent ACE inhibitory activities, with an IC_50_ value of 0.49 g/m^3^ when treated with Alcalase [95]. Similarly, peptides from *Chlorella pyrenoidosa* have exhibited an IC_50_ value of 0.21 mg/mL for the sequence FLKPLGSGK when treated with pepsin, and it was explained that peptides may interact with the active site of ACE, distorting the tetrahedrally coordinated Zn(II) ion and causing a loss of ACE activity [96]. Microalgae have been effectively used to create peptides with antihypertensive, antioxidant, and angiotensin-converting enzyme inhibitory qualities—all of which are critical in reducing the risk factors for cardiovascular disease (CVD) [97].

Diabetes mellitus is a growing concern, and algal proteins have shown promise in managing this condition. Bioactive peptides derived from algae have demonstrated the ability to inhibit DPP-IV, α-amylase, and α-glucosidase, enzymes involved in glucose metabolism. For example, peptides from *Arthrospira platensis* have shown DPP-IV inhibitory.

Activity with an IC50 value of 5.257 mg/mL when treated with Alcalase [98]. Another medical puzzle that needs to be solved urgently is cancer. Algal proteins and peptides have exhibited anti-tumor activities. For instance, a peptide sequence YGFVMPRSGLWFR from papain hydrolysates of *Arthrospira platensis* displayed anti-tumor activities on A549 cancer cells with an IC_50_ of 104.1 μg/mL [99]. Additionally, a peptide from *P. yezoensis*, PPY, has been shown to lower the expression of insulin-like growth factor I receptor (IGF-IR) binding proteins and inhibit the PI3K/Akt pathway, which are crucial in cell growth and proliferation of MCF-7 breast cancer cells [100].

Anti-inflammatory properties are linked to the modulation of pro-inflammatory cytokines, including IL-6 and TNF-α, and inhibition of the NF-κB signaling pathway, thereby attenuating inflammatory responses. The mechanisms of anti-tumor are complicated. Some peptides can trigger apoptosis in cancer cells by activating caspases and causing cytochrome c release from mitochondria, or arrest the cell cycle at specific phases, preventing cancer cells from proliferating [101]. It was also proved that peptides can block voltage-gated sodium channels or disrupt microfilaments and microtubules, inhibiting cancer cell proliferation, invasion, and metastasis [102]. The application of algae and algal-derived products in the pharmaceutical industry is displayed in Table 3.

While in vitro studies have demonstrated promising bioactivities, systematic in vivo validation and well-designed human intervention studies remain limited. Such studies are essential to confirm physiological relevance, bioavailability, safety, and effective intake levels of algal-derived proteins and peptides within the context of normal dietary consumption. The current scarcity of in vivo and clinical evidence partly reflects the complexity of translating in vitro findings into whole-body systems, where digestion, absorption, metabolic transformation, and interactions with the gut microbiota may substantially influence bioactivity.

Future research should therefore emphasize mechanism-based in vivo studies using appropriate nutritional or disease-relevant models, along with dose–response evaluation and biomarker analysis, to bridge the gap between molecular mechanisms and functional outcomes. In addition, well-controlled human dietary intervention studies are needed to establish scientifically grounded recommendations for the development of functional foods and nutraceutical products. Strengthening this evidence base will be crucial for advancing the practical application of algae-derived proteins and peptides in food and nutrition science.

### 3.3. Cosmetics Industry

#### 3.3.1. Skincare Formulations

Algae-derived peptides have exhibited activity in scavenging free radicals, anti-inflammatory properties, inhibiting skin aging enzymes, and protecting against UV-induced damage. These peptides have been found to improve skin conditions, reverse facial aging, and maintain skin elasticity, presenting promising opportunities for developing innovative skincare products in the cosmetic industry.

In recent years, microalgae proteins and peptides have garnered significant attention in the cosmetics industry for their anti-aging properties and UV protection. For instance, peptides derived from *Chlorella pyrenoidosa* have been shown to inhibit UVB-induced MMP-1 expression in skin fibroblasts by suppressing the activity of AP-1, CYR61, and MCP, thereby reducing collagen degradation and mitigating photoaging [115]. Similarly, fucoidans from brown algae such as *Costaria costata* exhibit anti-aging potential by suppressing UVB-induced upregulation of MMP-1 and downregulation of type I procollagen in human foreskin fibroblasts and HaCaT cells [116]. Additionally, peptides from microalgae can enhance skin hydration by increasing the expression of aquaporin-3 (AQP3) and hyaluronic acid synthase-3 (HAS3), which are essential for maintaining skin moisture [117]. Study also stated that some peptides derived from *Chlorella pyrenoidosa* act as tyrosinase inhibitors, reducing melanin synthesis and achieving skin lightening [116], while *Dunaliella salina* extracts were proven to exhibit potential in downregulating the expression of a key regulator of melanogenesis, microphthalmia-associated transcription factor (MITF), thereby suppressing melanin production [118].

There is a growing interest in leveraging the bioactive properties of microalgae proteins and peptides in skincare formulations for the cosmetic industry. However, several challenges remain. The production of microalgae-derived bioactives is still limited by high costs and scalability issues, and the use of genetically modified organisms (GMOs) in cosmetic products raises consumer concerns [117]. In the future, advancements in biotechnology and industrial fermentation are expected to address these challenges.

#### 3.3.2. Wound Healing and Skin Regeneration

Despite there are few applications in industry on wound healing of algae-derived proteins and peptides, some laboratory studies highlighted the relative activities, including stimulating fibroblast and keratinocyte proliferation, enhancing collagen synthesis, and modulating inflammatory responses, and so on. *A. platensis* extracts have been shown to accelerate wound healing by increasing collagen production and activating key signaling pathways such as Akt, ERK, and TGF-β1, which promote fibroblast proliferation and tissue repair [119]. Additionally, marine pectin derived from *Spirulina maxima* has been found to enhance cell proliferation and reduce wound area in both in vitro human dermal fibroblast models and in vivo zebrafish models [119]. In an excisional wound rat model, *A. platensis* gel and its nanoformulation significantly improved re-epithelialization, granulation tissue formation, and collagen content compared to untreated controls [120]. Most of the studies have been focused on *A. platensis* so far, and further clinical validation for other microalgae species is needed. Future directions may focus on optimizing microalgae-based formulations for targeted delivery, enhancing their bioavailability, and exploring novel microalgae species with untapped bioactive potential. Additionally, the development of photosynthetic scaffolds and bioactive hydrogels could further revolutionize wound care and tissue engineering [121].

## 4. In Silico Tools for Protein and Peptide Analysis

**Figure 2:**
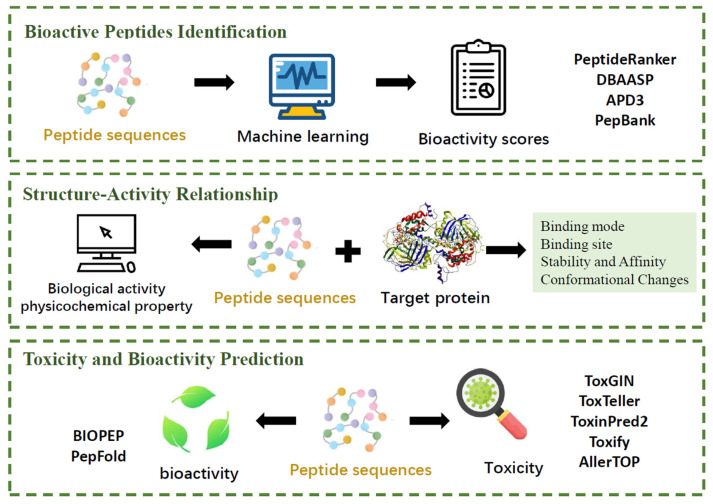
Application of in silico tools in algae-derived proteins and peptides analysis.

In silico methods are critical in the investigation of algae-derived peptides, making it easier to identify and characterize bioactive molecules with potential health benefits. Computational approaches advance our understanding of peptide characteristics, bioactivity, and mechanism of action, opening the door for their use in nutraceuticals and functional foods. Application of in silico tools in algae-derived proteins and peptides analysis refers to bioactive peptides identification, structure-activity relationship exploration and toxicity and bioactivity prediction, as present in Figure 2.

### 4.1. Identification of Bioactive Peptides

Computer technologies can greatly improve the identification of bioactive peptides. Recently, more and more studies have highlighted the power of computer technology in predicting and identifying bioactive peptides, which are subsequently generated and tested for biological activity. PeptideRanker is a useful tool for assessing the bioactivity of peptides that uses a machine learning algorithm to predict and sequence peptide activities. It facilitates the screening and prediction of new active peptides among a vast number of known active peptides, hence speeding up drug discovery and biological research. PeptideRanker was used in research that obtained high scores for peptides from red macroalgae, indicating their potential as ACE and DPP-IV inhibitors [122]. In silico frameworks were developed to mine bioactive peptides from annotated proteomes, such as *Chlorella vulgaris* and *Aphanizomenon flosaquae*, in order to find effective alpha amylase inhibitors [122]. A combination of enzymatic treatment, in silico analysis, and chemical synthesis was utilized to identify bioactive peptides from *Nannochloropsis oculate* [123]. Mass spectrometry was employed to determine peptide sequences, and in silico analysis was used to predict their bioactivities, including ACE-1 inhibitory activity and antithrombotic and CAMKII inhibition properties. This approach allowed them to highlight the potential of *Nannochloropsis oculata* biomass as a source of both protein and bioactive peptides for functional foods and feeds. Pedroni et al. developed an in silico framework to mine bioactive peptides from annotated proteomes, focusing on pancreatic alpha amylase inhibitory peptides from algae and cyanobacteria, including bioinformatics and molecular modeling to identify and predict the inhibitory potential of peptides [124].

For the identification of bioactive peptides, in silico methods not only save time and resources but also provide a systematic approach to uncovering the potential health benefits of various biological sources. By leveraging computational tools, researchers can efficiently screen and select peptide candidates for further experimental validation, thereby accelerating the discovery of novel bioactive compounds for pharmaceutical, food, and nutritional applications.

### 4.2. Structure-Activity Relationship

The application of computational techniques in studying the structure-activity relationships (SAR) of proteins and peptides is a pivotal approach in modern bioinformatics. These methods allow researchers to predict the biological activity of peptides based on their structural properties without the need for extensive in vitro or in vivo testing.

One of the primary computational tools used is quantitative structure-activity relationship (QSAR) modeling. QSAR involves the development of mathematical models that correlate the chemical structure of peptides with their biological activity. Commonly used software for QSAR analysis includes the OECD QSAR Toolbox, VEGA, etc. For instance, QSAR was used to understand the link between peptide structures and their activity towards different biological targets, such as ACE-1 inhibition [125]. Another computational method is molecular docking. This technique simulates the interaction between a peptide and a target protein, providing insights into the binding affinity and mechanism of action. Molecular docking can predict how a peptide will bind to a specific site on a protein, which is crucial for understanding its potential biological effects. Molecular docking can be achieved by AutoDock Vina v.2, 3D-DOCK FTDock v.2, HawkDock v.2‌, SwissDock 2024, and so on. In the study by Auwa et al. [126], molecular docking was used to understand the action of peptides working within the domains of angiotensin-converting enzyme (ACE). Computational simulations help identify binding sites and optimize peptide activity, as demonstrated with the peptide FDGIP from *Caulerpa lentillifera*, which showed significant antidiabetic properties [28].

In summary, computational techniques such as QSAR and molecular docking offer a way to predict and understand the biological activity of peptides, which is essential for the development of new therapeutics and functional foods. The use of these methods is particularly beneficial in the context of algae-derived peptides, where the potential for novel bioactive compounds is high, but the experimental validation can be resource-intensive.

### 4.3. Toxicity and Bioactivity Prediction

Toxicity predictions were used to ensure that identified peptides are safe for consumption, and advanced tools can achieve high accuracy in distinguishing between toxins and non-toxins [127]. By incorporating sequence similarity and evolutionary relationships, machine learning models were established to enhance prediction accuracy. ToxGIN is a model for predicting peptide toxicity using graph isomorphism networks, integrating amino acid sequences and 3D structural information. ToxTeller utilizes multiple machine learning models (logistic regression, SVM, random forests, XGBoost) to predict peptide toxicity, achieving high accuracy through a comprehensive dataset of toxic and non-toxic peptides [128]. ToxinPred2 and Toxify have shown accuracy as high as 95% in distinguishing toxins from non-toxins by a consensus model [129]. AllerTOP predicts allergenic properties. The toxicity of identified peptides derived from two red macroalgae (*Sphaerococcus coronopifolius* and *Gelidium spinosum*) was investigated, illustrating that no toxic effect was found. This indicates that the peptides are safe for potential use in food and nutraceutical applications [130].

As for bioactivity prediction, tools like BIOPEP were commonly used. The BIOPEP database serves as a comprehensive repository of biologically active peptide sequences and offers a suite of tools for analyzing their potential biological activities. It has become a standard tool in food peptidomics research, integrating bioinformatics and cheminformatics to support both in silico predictions and laboratory experiments [131]. BIOPEP was applied to highlight the potential health benefits, including antioxidant and anti-hypertensive activities of algal-derived peptides. Additionally, it has been used to predict the bioactivity of peptides derived from algae through in silico digestion, which helps in identifying proteins with greater potential for yielding bioactive peptides [132]. Overall, BIOPEP facilitates the discovery and characterization of novel bioactive peptides from algae, contributing to the development of functional foods and pharmaceuticals

Despite in silico tools significantly enhancing the efficiency of peptide analysis, in silico methods such as quantitative structure–activity relationships and molecular docking have not been extensively applied to the study of algal peptides, for their application to algae-derived peptides presents unique challenges. Algal proteins often exhibit high heterogeneity in amino acid composition, extensive post-translational modifications, and complex tertiary structures, which can limit the predictive accuracy of in silico hydrolysis and docking simulations [131]. For instance, QSAR models trained on terrestrial proteins may poorly extrapolate to marine algal sequences due to differences in residue frequency, hydrophobicity, and charge distribution. Similarly, molecular docking and dynamics approaches face difficulties in accurately modeling the flexibility and solvent interactions of algal peptides, particularly sulfated or glycosylated forms that are common in macroalgae [132]. Furthermore, machine learning models rely heavily on high-quality experimental datasets, which remain scarce for algal peptides, thereby constraining model reliability and generalizability. These factors collectively highlight the need for algae-specific parameterization, careful validation against experimental hydrolysis and bioactivity data, and the integration of multi-scale computational approaches to improve the predictive power and practical relevance of in silico studies for algae-derived bioactive peptides.

To address these challenges, an iterative integration of computational predictions with targeted experimental validation is recommended. Initially, in silico methods such as virtual enzymatic hydrolysis, QSAR modeling, molecular docking, and machine learning are employed to screen and prioritize peptide candidates based on predicted bioactivities and stability. Selected candidates are then subjected to targeted experimental validation, including enzymatic hydrolysis, peptide purification, and bioactivity assays, to confirm predicted functions. Feedback from experimental results can subsequently refine computational models, improving their accuracy and predictive power for algal sequences. This iterative strategy maximizes efficiency, reduces resource consumption, and ensures that computational predictions are grounded in empirical evidence.

## 5. Challenges and Opportunities in Algae-Derived Proteins and Peptides

**Figure 3:**
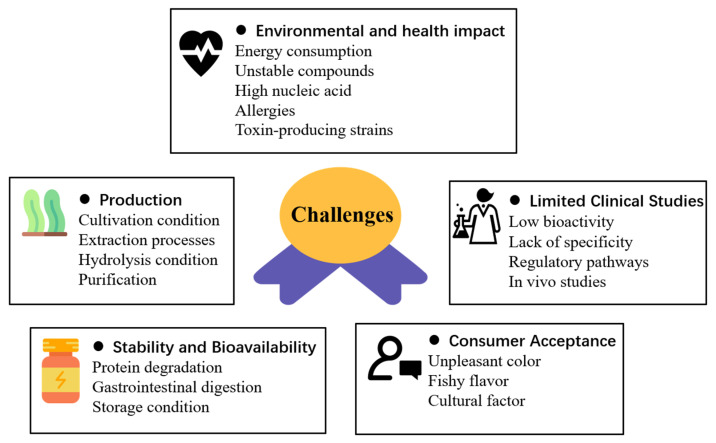
Challenges of algae-derived proteins and peptides application.

Despite their promising nutritional and therapeutic potential, the translation of algae-derived proteins and peptides into large-scale applications is far from straightforward. A range of technical, biological, environmental, and socio-economic challenges constrain their scalability and practical use, as displayed in Figure 3. Nevertheless, each of these challenges also provides opportunities for innovation. The following subsections provide a critical discussion of these challenges and opportunities, with a focus on production, clinical validation, stability, environmental and health impacts, and consumer acceptance.

### 5.1. Production

The production of macroalgae is primarily limited to a few European countries, with 68% of macroalgae harvesting still relying on wild stocks [133]. The production of algae-derived proteins and peptides presents a unique set of challenges that can impact scalability and sustainability. Firstly, algae cultivation and growing conditions need to be optimized to maximize yield. That would be affected by a series of factors, such as light, temperature, and nutrient availability. Additionally, the extraction processes can be complex and costly; advanced technologies or optimized enzymatic treatments are required to ensure product quality and functionality. Proteins from resilient algal cell walls, which contain about 70% of the protein content, are difficult to access. In order to accelerate enzyme reactions, *Chlorella microphylla* has been subjected to ball-milling cell lysis to get around concentration limitations [134]. The effectiveness of enzymatic hydrolysis depends on the availability of particular cleavage sites for each enzyme [135], and the selection of enzymes is very important. For example, pepsin possesses endonuclease activity, which breaks internal peptide bonds, resulting in bigger fragments. This method is more efficient than relying just on pancreatic enzymes or a combination of the two [136]. Moreover, the polysaccharide content of the microalgae cell wall hinders protein extraction, affecting protein yield, purity, and digestibility [137]. Finally, the processes of purifying can be complex and may result in the loss of activity or integrity of the peptides due to the harsh conditions required for separation and purification, such as high temperatures or extreme pH levels [4,138].

In addition to technological limitations, biological factors also influence the large-scale production of microalgal proteins. Species selection plays a critical role in determining protein yield, amino acid composition, growth rate, and environmental adaptability. Biomass variability arising from cultivation conditions, seasonal fluctuations, and strain-specific metabolic responses may further affect compositional consistency and downstream processing efficiency. Moreover, large-scale cultivation systems remain vulnerable to microbial contamination and competition, which can compromise productivity and quality control. The exploration of unconventional or region-specific algal species, including invasive biomass streams, may offer opportunities for sustainable resource utilization; however, careful ecological and safety evaluation is required to ensure environmental compatibility and regulatory compliance. Addressing these biological and ecological considerations will be essential for improving process robustness and scalability.

### 5.2. Limited Clinical Studies

Algae-derived proteins and bioactive peptides exhibit a wide range of health-promoting properties, particularly in their antioxidant, anti-hypertensive, anti-diabetic, and anti-cancer activities. Proteins and bioactive peptides, derived from sources like *Chlorella* sp. and *Arthrospira platensis,* have garnered attention for applications in nutraceuticals and pharmaceuticals [139]. Their low molecular weight and specific amino acid compositions enhance their bioactivities, making them valuable for alleviating oxidative stress, cardiovascular diseases, cancer, and diabetes. However, while algae proteins and peptides show promise for clinical efficacy, there are still hurdles to overcome in terms of low bioactivity, lack of specificity, ensuring consistent quality, and navigating regulatory pathways for clinical use [140].

In the field of cancer treatment, the narrow therapeutic window of conventional chemotherapy agents is exacerbated by their inability to selectively target cancer cells, causing toxicity to healthy tissues. While some peptides show high affinity and specificity in identifying cancerous and healthy cells, more algae-derived proteins and bioactive peptides face limitations in this regard as traditional chemotherapeutic agents, leading to side effects in cancer treatment [141]. Research stated that many peptide drugs target cell surface receptors that are overexpressed in cancer cells, but these receptors can also be present in healthy cells, leading to unintended effects [142]. Moreover, most of the studies that showed promising results in peptide bioactivity were conducted in vitro, but clinical studies in vivo were more important to validate the health benefits of algal proteins and peptides. There is a need to state the safety, efficacy, and optimal dosages of algal proteins and peptides in humans.

### 5.3. Stability and Bioavailability

Many algal bioactive compounds are sensitive to environmental conditions, leading to degradation and loss of beneficial properties. The bioactivities and bioavailability of algae peptides are significantly influenced by biological, chemical, and physical processing methods, as well as storage conditions, by altering their structure, which may affect their functional properties and mechanisms of action [143]. These factors induce inherent changes that either enhance or diminish the functional properties of the peptides. Enzymatic hydrolysis improves the digestibility and bioactivity of microalgae and cyanobacteria proteins. yielding bioactive compounds with antioxidant and antimicrobial properties [144]. A study showed that antioxidant activities of protein hydrolysates remain stable under various heat treatments, indicating resilience to processing [144], but there is an opposite statement that traditional processing techniques, including heating, may negatively affect the peptides’ chemical properties, impacting their activity and availability. Moreover, the clinical application of marine algal peptides is hindered by poor bioavailability, which affects their absorption and utilization in the body, limiting their therapeutic effectiveness in cancer treatment [142].

The stability of algae-derived peptides is influenced by food processing, gastrointestinal digestion, and interactions with food matrices. Thermal, mechanical, or chemical stresses during processing can reduce peptide bioactivity, which can be mitigated by microencapsulation or coacervation using proteins and polysaccharides [142]. Storage condition is an important topic that needs to be considered when studying the application of microalgae peptides. Optimal storage at −20 °C and neutral pH (7) maintains the stability of bioactive peptides from *Macrocystis Pyrifera,* while extreme conditions can lead to degradation [145]. To maintain the stability and bioavailability, innovative delivery systems, such as nanocarriers, are being explored to ensure controlled release and prolonged therapeutic effects [114]. During digestion, low pH and enzymatic hydrolysis may degrade peptides, whereas controlled-release formulations help preserve bioactivity and facilitate intestinal absorption [146]. Interactions with other food components, such as proteins, lipids, and polysaccharides, can either stabilize or hinder peptide function, and careful optimization of formulation parameters, including pH, ionic strength, and water activity, is essential. Overall, encapsulation strategies and matrix design play key roles in maintaining the stability and functional properties of algae-derived peptides in functional food applications.

Microencapsulation technology has been applied to algae-derived proteins and peptides in food products to protect their bioactive properties, improve stability, and mask undesirable flavors and odors. This technique involves encapsulating the sensitive proteins and peptides in protective coatings, allowing their controlled release and enhancing their functionality in various food matrices. A study showed that *spirulina* protein encapsulated with gum arabic and maltodextrin retained significantly higher protein content and antioxidant activity under high temperature and humidity conditions [147]. Microencapsulated peptides from *Spirulina* and *Chlorella* were applied in beverages and dairy products to maintain functionality and stability [148]. Beyond these examples, recent studies have explored different wall materials such as whey protein, pectin, and alginate, which can modulate release kinetics and improve resistance to thermal and mechanical stresses during processing. Co-encapsulation with natural antioxidants or prebiotics has also been reported to synergistically enhance peptide stability and preserve bioactivity. Moreover, advanced techniques like spray-drying, freeze-drying, and coacervation have been optimized to maximize encapsulation efficiency and functional retention, demonstrating the versatility of microencapsulation in supporting the integration of algae-derived peptides into diverse food formulations.

### 5.4. Environmental and Health Impacts

The exploration of algae as a source of bioactive peptides offers numerous benefits, including their potential as sustainable protein sources and their health-promoting properties. However, the large-scale production and processing of algal biomass for peptide extraction also present certain challenges to the environment. For instance, a paper highlighted the need for sustainable protein extraction processes, which must be optimized to be economically viable and environmentally friendly [149]. The use of physical and biochemical pre-treatments, as well as emerging technologies like subcritical water processing, must be carefully managed to minimize energy consumption and waste production. Additionally, current production methods may not yet be optimized for large-scale commercialization, which could lead to increased resource use and environmental impact if not addressed [150]. Furthermore, the review discussed the influence of biotic and abiotic factors on algal metabolites [151], indicating that changes in environmental conditions due to human activities could affect the quality and quantity of bioactive compounds produced by algae, potentially impacting their sustainability as a resource.

On the other hand, algae application may cause a negative effect on human health. Nowadays, algal biomass is more commonly used than algal proteins and peptides in foods. High content of nucleic acid in algal biomass can increase uric acid, potentially leading to gout and kidney stones, highlighting the need for strategies to reduce nucleic acid content [152]. Moreover, it is a known fact that proteins from various sources can cause allergies in some individuals. Although hydrolysis and peptide purification generally reduce allergenic potential, residual epitopes may still elicit reactions. Since algae proteins are a novel source of bioactive compounds, there could be a risk of allergic reactions in sensitive individuals. A study stated that unique proteins and polysaccharides within certain algal strains raise concerns about allergic reactions [153]. Some microalgae proteins may trigger an immune response by the immunoglobulin E (IgE) antibody and are classified as allergens [153]. Computational tools for allergenicity prediction, combined with in vitro immunoassays and, where necessary, animal studies, can help evaluate potential risks before human consumption. Careful documentation and labeling are also required to inform consumers and comply with food safety regulations, particularly when introducing novel algal peptides into functional foods or nutraceutical products.

The paper also pointed out that the presence of metabolites in algae depends on biotic and abiotic factors. This suggests that the composition and properties of algae-derived products can vary, which might lead to inconsistent effects on human health, including negative outcomes. Safety assessment is a critical step in the development of algae-derived peptides for food applications. Potential risks include contamination with heavy metals, toxins, or microbial pathogens originating from the algal biomass, as well as unintended bioactive effects of novel peptides [151]. Standard safety evaluation strategies involve chemical and microbiological testing of the raw material, in vitro cytotoxicity assays, and preclinical in vivo studies to assess potential adverse effects. Regulatory guidelines, such as those established by the European Food Safety Authority (EFSA) or the U.S. Food and Drug Administration (FDA), recommend a tiered approach to safety assessment, ensuring that peptides intended for human consumption meet established safety thresholds.

### 5.5. Consumer Acceptance

The application of algae proteins and peptides in food products is facing challenges due to taste and consumer acceptance. It was found that crackers added with certain algae species, such as *Palmaria palmata*, *Saccharina latissima*, and green *Spirulina*, were less liked by consumers compared to those with *Lithothamnium calcareum* and blue *Spirulina*. The crackers with the former algae were described with negative sensory attributes like “off-flavor”, “fishy flavor”, and “bitter”, which contributed to their lower acceptance [154]. Protein hydrolysates are associated with a bitter taste, which could be a problem for oral consumption. To go further, acceptance of algal foods is influenced by cultural factors. For instance, Italians exhibited lower food neophobia, leading to higher acceptance of Spirulina [154]. Younger consumers and those concerned with sustainability are more likely to accept microalgae-based products, emphasizing the importance of taste in product development [155]. The taste and specific sensory properties of algae-based products can significantly impact consumer acceptance; thus, the successful application of algae proteins and peptides in food products requires careful consideration of these factors to enhance palatability and market appeal.

## 6. Future Perspectives

The burgeoning research on microalgal proteins and peptides over the past decade has illuminated their vast potential across diverse fields, including nutrition, pharmaceuticals, and cosmetics. However, several key areas remain underexplored and present promising avenues for future research. One critical direction is the optimization of large-scale production processes for microalgal biomass and protein extraction. Current methods are often hindered by high costs and complex extraction protocols, which limit the economic viability and scalability of microalgal protein production. Future studies should focus on developing more efficient and sustainable techniques, such as advanced enzymatic treatments and integrated bioprocessing systems, to enhance yield and purity while minimizing environmental impact. Additionally, it is important to note that their translational and clinical relevance, particularly in the context of food applications, remains to be fully established. Factors such as peptide stability during digestion, bioavailability, potential interactions with food matrices, and safety profiles need to be carefully evaluated before drawing conclusions about functional benefits in humans. there is a need for comprehensive clinical trials to validate the health benefits of microalgal peptides.

Another emerging area of interest is the development of innovative delivery systems to improve the stability and bioavailability of microalgal peptides. Techniques such as microencapsulation and nanoparticle formulation have shown promise in protecting peptides from degradation in the gastrointestinal tract and enhancing their absorption across the intestinal barrier. Further research should explore the potential of these delivery systems in various applications, including functional foods, nutraceuticals, and pharmaceuticals.

Importantly, as research progresses toward practical application, regulatory considerations will increasingly influence the translation of microalgal peptides into functional food and nutraceutical markets. In many regions, newly developed algal-derived ingredients may fall under novel food frameworks or require formal safety substantiation before commercialization. Therefore, future research should incorporate compositional characterization, intake estimation, and safety evaluation strategies alongside bioactivity assessment, allowing regulatory requirements to be considered early in the research design phase. Such integration will facilitate smoother market entry and strengthen the real-world feasibility of these bioactive ingredients. By addressing these challenges and exploring these opportunities, future research can unlock the full potential of microalgal proteins and peptides, paving the way for innovative solutions in health, nutrition, and biotechnology.

## 7. Conclusions

The exploration of microalgal proteins and peptides represents a rapidly advancing frontier in sustainable biotechnology, with significant potential in nutrition, health, and functional ingredient development. Although substantial progress has been made in identifying antioxidant, antihypertensive, antimicrobial, and immunomodulatory activities, the field remains largely centered on in vitro screening and species-specific studies, with limited methodological standardization and translational integration. Future advancement requires a shift from descriptive bioactivity reporting toward an integrated, data-informed development framework. Beyond optimizing production scalability and bioavailability, greater emphasis should be placed on harmonized evaluation strategies and regulatory alignment. Importantly, the integration of experimental validation with computational approaches, such as molecular docking, QSAR modeling, and machine learning-assisted peptide prediction, offers a pathway to transition from empirical screening to rational and efficiency-driven discovery. By coupling mechanistic understanding with scalable production and digital innovation, microalgal proteins and peptides are poised to evolve toward precision-guided and industry-oriented development, strengthening their contribution to sustainable food systems and next-generation bio-based products.

## Figures and Tables

**Table 1:** The protein content and digestibility of different algae species.

	Protein (% dw)	Protein Content	EAA (% TAA)	Digestibility
Red algae	7–47% [15]	*P*. *palmata* 12.5% [18]; *C*. *crispus* 21% [19]; *P*. *dioica* 28.7% [20]	*O*. *pinnatifida* 41.62% [21]; *P. umbilicalis* 42.4% [22]; *P. palmata* 47% [23]	*P. palmata* 87.3% [24]; *P. columbina* 74.3% [25]; *S*. *gaudichaudii* 86.7% [26]
Green algae	9–33% [17]	*U*. *lactuca* 8.7–32.7 [27]; *C*. *rupestris* 29.8% [27]; *C*. *lentillifera* 19.38% [28]	*C*. *vulgaris* 33.2% [16]; *D*. *salina* 42.45% [16]; *U. australis* 42% [26]; *U. intestinalis* 40% [26]	*U. australis* 66.6% [29]; *U*. *lactuca* 85.7% [30]
Brown algae	3–24% [17]	*A. nodosum* 3%, *A. esculenta* 9–20%, *Fucus* spp. 3–11% [31]; *U. pinnatifida* 24% [29]	*P*. *carterae* 45.85% [16]; *Fucus spiralis* 63.5% [21]; *U. pinnatifida* 48.4% [22]; *H. elongata* 47% [22]	*U. pinnatifida* 48.1% [22]; *S*. *fusiforme* 51.8% [24]; *E. bicyclis* 57.1% [32]
Cyanobacteria	2.5–73.7% [17]	*R. bullata* 19.83%, *N. pongiaeforme* 6.15% [31]; *D. tharense* 28.1%, *A. variabilis* 34.4% [28]; *A. Paracas* 73.7% [33]	*A. platensis* 42%, *Nostoc* sp. 44.72% [16]	-

**Table 2:** The application of algae and algal-derived products in food industry.

Application	Species	Algal Product Added	Food Product	Protein Increasing	Other Nutrients Increasing	Physico-Chemical Characterization	Sensory	Bioactivity	Reference
Nutrition-promoting component	*S*. *platensis*	Biomass powder (1% and 2%)	Bread	3.17% and 5.12%	EAA (0.06% and 0.22%)	-	-	-	[73]
		Biomass powder (0.25%, 0.5% and 1%)	Cheese	1.5%, 4.8% and 9.8%	Fat (1.8%, 4.4% and 8.7%)	-	0.25% and 0.5% were mostly preferred	-	[74]
		Microencapsulated biomass powder (1%)	Ice cream	From 35% to 53%	Lipid (5.5% to 22.2%)	Encapsulants accelerate melting	Acceptability index superior than 70%	-	[75]
	*S*. *maxima*	Microencapsulated Biomass powder (20%)	Biscuits	40%	Higher unsaturated fatty acid ratio	-	Acceptability index 79.2%	-	[76]
	*A. platensis*	Biomass powder (4%)	White chocolate	23.1%	TAA (45%), lipids (10.3%), minerals (13.5%)	-	No significant (*p* > 0.05) difference	-	[77]
	*Spirulina* spp.	Biomass powder (2.6%)	Rice flour and corn flour snacks	22.6%	Lipids (28.1%) minerals (46.4%)	Affecting the expansion index and hardness (*p* > 0.05)	Acceptability index 82%	-	[78]
	*Chlamydomonas* spp. and *Nannochlo* spp.	Biomass powder (3%)	Gluten-free bread	-	-	Mixing properties were not affected	Higher sensory score	-	[79]
	*I*. *galbana*	Biomass Powder (2%) and ethyl acetate lipidic extract (2%)	Yoghurt	25%	ω3 LC-PUFA (60 mg/100 g), DHA (9.6 mg/100 g)	-	-	-	[80]
Food colorant	*P*. *cruentum*	B-phycoerythrin (1.6 mg/L to 49.5 mg/L)	Milk-based products	-	-	Stable in pH 4.0 to 9.0; stable in 11 days of cold storage	Pink color simulating	-	[81]
	*S*. *platensis*	Phycocyanin (0.5%,1%,1.5%)	Yoghurt	0.9–3.1%	Ash (0.8–13.5%)	-	Higher sensory score	-	[82]
	*A. platensis*	C-phycocyanin (15.6–111.7 mg/L)	Beverages	-	-	Stable in pH 3.0–9.0; stable in 11 days of cold storage	Blue color simulating	-	[63]
	*U*. *Lactuca and* *G*. *verrucosa*	Chlorophyll (45%), carotenoids (31%) and phycobiliprotein (33%)	Jell dessert	-	Ash	30 days with 70% color retaining	Acceptability index over 70%	-	[83]
Functional food ingredients	*Tetraselmis* spp., *Spirulina* spp., *Chlorella* spp.	biomass powder (0.5–2.0%)	Broccoli soup	-	Polyphenols (32.9 to 45.6 mg/100 mL)	-	Acceptability index over 70%	Antioxidant	[71]
	*Spirulina* spp.	Biomass powder (5.0% and 8.75%)	Chocolate milk powder	8.8% and 25.0%	Phenolic (31% and 39%); fat decreasing (33.6% and 34.9%)	Good suspension stability and low hygroscopicity	No significant (*p* > 0.05) difference	Antioxidant	[84]
	*Spirulina* spp.	Biomass powder (2–4%)	Sauce	34%	Minerals (180–356%)	Stable in 45 days; Lower cohesiveness and higher elasticity	No significant (*p* > 0.05) difference	Antioxidant	[85]
	*Chlorella* spp.	Biomass powder (3.5%)	Grissini	-	Total phenolic content (TPC); essencial amino acid (EAA)	No texture differences	Acceptable sensorially	Antioxidant	[86]
	*Spirulina* spp.	Biomass powder (1.5%)	Cracker	-	Total phenolic content (TPC); essencial amino acid (EAA)	Hardness decreased	69% of the participants acceptability index over 70%	Antioxidant	[86]
	*C*. *vulgaris*; *A*. *nodosum*	Biomass powder (0.5–2.0%)	Vegetable cream	Higher protein content	Iodine value	-	Acceptability index over 70%	Antioxidant	[72]

**Table 3:** The application of algae and algal-derived products in the pharmaceutical industry.

Bioactivity	Compounds	Species	Therapeutic Effect/Mechanism	
Antioxidant	GMNNLTP (peptide)	*N*. *oculata*	Inhibiting ACE activity	[103]
	MMLDR (peptide)	*S*. *maxima*	Suppressing cytokine generation by endothelial cells	[103]
	Bioactive peptides	*G*. *lemaneiformis*	Scavenging radical; metal ion chelation; activating Nrf2/HO-1 signaling; stabilizing mitochondrial membrane potential	[104]
	Bioactive peptides	*C*. *vulgaris*	Scavenging ROS and inhibiting lipid peroxidation	[105]
	Bioactive peptides	*G*. *gracilis*	ROS scavenging	[106]
Antihypertensive	TGAPCR and FQINCILR (peptides)	*G*. *lemaneiformis*	Reducing systolic blood pressure (SBP) and diastolic blood pressure (DBP) in Spontaneously hypertensive rats	[107]
	Protein hydrolysates	*M*. *japonica*	Inhibiting ACE activity	[108]
	Bioactive peptides	*G*. *gracilis*	Inhibiting ACE activity	[106]
Antidiabetic	Protein hydrolysate	*P*. *palmata*	Inhibiting DPP-IV and upregulating insulin, GLP-1 and GIP secretion in vitro	[109]
	Protein hydrolysate	*P*. *palmata*	Reducing food intake and improving beta-cell function in vivo	[110]
	Bioactive peptides	*A. platensis*	Competitively inhibiting the DPP-IV active site; bonding to hydrogen and hydrophobic interactions with catalytic residues	[111]
	Bioactive peptides	*Spirulina* spp., *Chlorella* spp., *Nannochloropsis* spp.	Inhibiting ACE, DPP-IV, and renin that are structure–activity-driven; anti-inflammatory effects	[112]
	Bioactive peptides	*G*. *gracilis*	inhibiting DPP-IV	[106]
Anti-inflammatory	Protein hydrolysates	*A. platensis*	Modulating inflammatory cytokines (decreasing TNF-α, IL-6), promoting fibroblast migration, stimulating collagen deposition; nanoliposomes enhancing skin penetration	[113]
Anti-cancer	Bioactive peptides	Various marine micro- and macroalgae	Inducing intrinsic apoptosis; cell-cycle arrest; modulating DNA-repair and senescence pathways; mitochondrial dysfunction	[114]

## Data Availability

The original contributions presented in this study are included in the article. Further inquiries can be directed to the corresponding authors.
